# Potent antibacterial, antioxidant and toxic activities of extracts from *Passiflora suberosa* L. leaves

**DOI:** 10.7717/peerj.4804

**Published:** 2018-05-30

**Authors:** Kumudu R.V. Bandara, Chayanika Padumadasa, Dinithi C. Peiris

**Affiliations:** 1 Department of Zoology (Centre for Biotechnology), University of Sri Jayewardenepura, Nugegoda, Western Province, Sri Lanka; 2 Department of Chemistry (Centre for Plant Materials & Herbal Products Research), University of Sri Jayewardenepura, Nugegoda, Western Province, Sri Lanka

**Keywords:** *Passiflora suberosa*, Antibacterial, Antioxidant, Toxic effects

## Abstract

*Passiflora suberosa* L. belonging to the family Passifloraceae is an important medicinal plant used in traditional medicinal system in Sri Lanka to treat diabetes, hypertension and skin diseases. We extracted *P. suberosa* leaves under reflux conditions using different solvents (hexane, chloroform, methanol and water), then subjected to phytochemical screening. Alkaloids, flavonoids and saponins and saponins and anthraquinones were present in hexane and chloroform extracts. Alkaloids, unsaturated sterols, triterpenes, saponins, flavonoids and tannins were observed in both methanol and aqueous extracts. Proanthocyanidins were observed only in the aqueous extract. Hence, aqueous and methanol extracts with most classes of phytochemicals present were subjected to antimicrobial, antioxidant, antihaemolytic activities and Brine shrimp lethality studies. Antibacterial activity and minimum inhibition concentrations were evaluated using three Gram-positive (*Bacillus subtilis, Staphylococcus aureus* and *Enterococcus faecium*) and three Gram-negative bacteria (*Pseudumonas aeruginosa, Salmonella typhimuriam* and *Escherichia coli*). The results indicated that only the methanol extract of *P. suberosa* exhibited antibacterial activities against all the strains of Gram-negative and Gram-positive bacterial with stronger activity against Gram-negative bacteria. DPHH (2,2-diphenyl-1-picrylhydrazy) scavenging assay was adopted to evaluate antioxidant properties while antihaemolytic and toxic activities were studied respectively using cow blood and Brine shrimp lethality assay. The IC_50_ values of the aqueous extract in both antioxidant and antihaemolytic assays were significantly lower than the standard ascorbic acid. Similar results were observed in the Brine shrimp lethality assay. In conclusion both aqueous and methanol extracts of *P. suberosa* leaves showed the presence of majority of phytochemicals including proanthocyanidins. Antibacterial activity was obtained only for methanol extract with better activity against Gram-negative bacteria. The aqueous extract showed better antioxidant, antihaemolytic and toxic activities than the methanol extract and their respective standards. Further investigations on the chemical composition and possible isolation of active ingredients is warranted.

## Introduction

Being rich bio-resource of drugs, for centuries plants derived from drugs have become an essential source of traditional medicine specially in the developing countries (Atanasov et al., 2015). Even today, the majority of the world’s populations still rely on complementary medicine owing to their ability to combat various human diseases (Rangika, Dayananda & Peiris, 2015; Peiris, Dhanushka & Jayatilleka, 2015). The active principle of plants are the phytochemicals, mainly secondary metabolites including tannins, flavonoids, terpenoids, saponins, cyanogenic glycosides, nitrogen compounds (alkaloids, amines, betalanines), phenols, and phenolic glycosides (Abdelwahab et al., 2010), which play a vital role in reinforcement of plant tissue and plant survival (Anwer et al., 2013) against free radicals, cell proliferation and pathogenic microorganisms (Obeidat et al., 2012).

The increasing resistance of bacteria to antibiotics poses a significant challenge when combating infectious diseases caused by bacteria. This is a major risk for the world population; for example, currently in the United States alone people are adversely infected with pathogenic bacterial strains demonstrating resistant against antibiotics prescribed to treat infections. Furthermore, about 23,000 deaths are recorded each year due to antibiotic resistant infections (Center for Disease Control and Prevention, 2013). Drug resistant microbes can fast spread between people and animal, and from person to person. Gram-positive bacteria including *Staphylococcus aureus * (causing a range of infectious diseases from skin and wound infections to phenumonia); * Enterococcus faecium*, which is a leading cause of multi-drug resistant bacteria responsible for infections due to central lines, urinary drainage catheters, and ventilators. Gram-negative bacteria including *Salmonella typhi* (causing typhoid fever); *Pseudomonas aeruginosa*, a multidrug resistant pathogen responsible for triggering healthcare-associated infections including pneumonia, bloodstream infections, urinary tract infections, and surgical site infections are some examples for drug resistant bacteria (Center for Disease Control and Prevention, 2016). The alarming rise of drug resistant bacteria are problematic to control with the current available antibiotics on the market. Discovery of new antibiotics via *de novo* synthesis are time-consuming and expensive. Hence, the search of new remedies is urgently required (Center for Disease Control and Prevention, 2013). Recently, novel antibacterial antidotes from natural derivatives have been described and thus plants are receiving extensive consideration as possible antibiotic agents.

Living cells in the human body continuously produce reactive oxygen species and free radicals and are controlled by body enzymes. Over production of either free radicals, reactive oxygen species or failure in the defense mechanisms result in severe damage to cells and tissues leading to non communicable chronic diseases, specifically cancer. Antioxidant compounds have the ability to scavenge these free radicals or reactive oxygen species, which contribute to the various etiology of chronic health problems. Laboratory research conducted with rats have shown that the artificial antioxidants in the market could cause internal and external bleeding (Poljsak, Suput & Milisav, 2013). Antioxidant activity can also be determined using erythrocyte antioxidant proficiency. Mammalian erythrocytes have their own morphological and biological characteristics. Hemoglobin molecules and accumulation of polyunsaturated fatty acids make erythrocytes more vulnerable for peroxidation. Mammalian erythrocytes are continuously exposed to high oxygen tension leading to oxidative damages (Babu, Shylesh & Padikkala, 2001). Further exposure to toxicants can result in generation of free radicals causing membrane damages and hemolysis (Chakraborty & Verma, 2010). Hence, attention have given to use of naturally occurring antioxidants such as flavonoids, polyphenolic compounds to scavenge free radicals to protect erythrocyte membranes (Kaur, Kapoor & Kaur, 2011).

*Passiflora suberosa* of the family Passifloraceae is a native plant to tropical America and have been introduced and naturalized throughout the tropics (Space & Flynn, 2000). Of the genus *Passiflora,* the species *suberosa* is a relatively unexploited plant compared to other species. This has promoted us to investigate its antioxidant and antibacterial potentials. This plant species is known vernacularly as wild passion fruit, devil’s pumpkin or indigo berry and has been traditionally used by physicians for hypertension, skin diseases (Vianna et al., 2012; Dhawan, Dhawan & Sharma, 2004) and as a sedative and antihypertensive remedy in Mexican folk medicine (Edgar et al., 2013). In previous studies, Sudasinghe & Peiris (2018), demonstrated hypoglycemic activities of aqueous leaf extract of *P. suberosa* in mice. Leaves from the plant is eaten as a green vegetable. In the present study, phytochemical screening was conducted for several extractions. Since the aqueous and methanol extracts showed promising results for the phytochemical screening, these two extractions were extended to investigate the antioxidant, cytotoxic and antibacterial potential using gram-positive and -negative bacterial strains.

## Materials & Methods

### Chemicals

All chemicals, and reagents were of AR grade and obtained from Sigma-Aldrich, Germany. Water when used was distilled using GFL distillation apparatus.

### Plant material and extraction

Fresh tender leaves of *P. suberosa* were hand-picked between 8.00 a.m.–9.00 a.m. from healthy plants from Nugegoda (6.4200°N, 80.0000°E), Sri Lanka. The plant was authenticated by Ms. S. A. H. P. Sudasinghe at Royal Botanical Garden, Peradeniya and a herbarium specimen (no: PS/01) was deposited. Leaves were washed with tap water, wiped and air-dried in the shade for 3 weeks. Dried leaves were cut into pieces, ground and stored in the freezer at −80 °C in sealed polythene bags until additional use.

Ground powder of *P. suberosa* leaves (100 g) were refluxed using 300 mL of hexane, chloroform, methanol and water separately for two hours. The resulting crude extracts were cooled to room temperature, filtered and made up to a final volume of 200 mL. Preliminary phytochemical screening was conducted to extract. Remaining portion of each extract was freeze dried for bioactivity testing. Freeze dried extracts were stored in the freezer −80 °C until further use.

### Phytochemical analysis

Secondary compounds present in hexane, chloroform, methanol and aqueous extracts of *P. suberosa* leaves were determined. To determine the presence of alkaloids, sterols, triterpenes, saponins, flavonoids, proanthocyanidins and anthraquinones, methods described by Harborne (1998) was adopted. Since flavonoids and saponins could interfere with results of each other, thin layer chromatography was conducted for both saponins and flavonoids. To confirm presence of flavonoids in both aqueous and methanol *P. suberosa* leaf extracts, 6.8:0.2:2.8:0.2 (ethyl acetate: formic acid: dichloromethane: methanol) solvent system was used. Spot development was observed under the UV light subsequent to the spraying of visualizing chemicals. Raw leaf powder was dissolved in distilled water and shaken for 30 s to observe froth formation indicating the presence of saponins. The color intensity or the precipitate formation was used as analytical responses to these tests.

### Antibacterial activity

Three Gram-positive bacteria species including *Bacillus subtilis*, *S. aureus* (ATCC25923) and *Enterococcus faecium* and 3 Gram-negative bacteria species including *Pseudumonas aeruginosa* (ATCC27853), *Salmonella typhimuriam* and *Escherichia coli* (ATCC 25922) was used in the study. The pure cultures of all the bacterial strains, which were selected on the basis of their importance as human pathogens except for the *B. subtilis*, which a model organism, was a gift from the Department of Microbiology, Faculty of Medical Sciences, University of Sri Jayewardenepura.

### Antibacterial activity by agar well diffusion assay

Muller-Hinton agar media were made and under aseptic condition, laid in sterilized disposable petri dishes. Over night culturing of bacteria was conducted at 37 °C in Muller-Hinton agar. Homogenized bacterial cell suspensions was adjusted to 0.5 McFarland standards (5  × 10^8^ CFU/mL). A disc impregnated with gentamycin (10 µg/disc) was used as a standard, while the respective solvents were used as the negative controls. The agar Petri dishes were incubated for 24 h at 37 °C. The antibacterial activity was evaluated by measuring the diameters of the growth inhibition zones (mm) for the tested pathogenic bacteria compared to the standards (Valgas et al., 2007). the measurement of the inhibition zones was conducted using three sample replications.

### Minimum inhibitory concentration for antibacterial activity

Strains exhibited inhibition zones were subjected to minimum inhibitory concentration test. The minimum concentrations of a plant extract are the concentration where no growth was observed in a solid medium. To determine the MICs, the plant extracts were serially diluted ranging from 6.25 to 800 µg/mL (Samie et al., 2010). Microplates prepares with respective medium was inoculate with 0.5 Mcfarland standard (10^8^ CFU/mL). Dilution series was prepared using broth micro-dilution procedure (1:100). Upon incubation at 37 °C for 1 day, the MIC was recorded (Otang, Grierson & Ndip, 2012).

### Antioxidant screening

Free radical scavenging capability of freeze dried methanolic extract of *P. suberosa* leaves was determined using the DPPH assay according to a previously published method upon modification (Blois, 1958). A concentration series (800–6.25 µg/mL) of the freeze dried methanolic extract was prepared in methanol and DPPH was prepared by dissolving 10 mg of DPPH in 250 mL of methanol. Sample solutions at various concentrations (1.5 mL) were mixed with DPPH solution separately and the resulting mixtures were allowed to stand at room temperature for 30 min. Extent of discoloration of mixtures were determined at 517 nm using the UV-Visible spectrophotometer. All measurements were carried out in triplicate. For the control and the blank, DPPH solution in methanol and methanol was used respectively. Ascorbic acid was used as the standard. DPPH scavenging activity of the extracts were expressed as IC_50_ values, and was calculated using the equation given below.

\begin{eqnarray*}\text{DPPH scavenging effect (%)}=1-(\text{absorbance of sample/absorbance of control})\times 100. \end{eqnarray*}

The same procedure was repeated for the aqueous extract of *P. suberosa* leaves.

### Antihaemolytic activity

The antihaemolytic activity of methanol and aqueous leaf extracts of *P. suberosa* was quantitatively analyzed using cow blood. Blood was collected from a slaughter house in Colombo and was centrifuged for 10 min at 1,500 rpm (Model: Minor 35, UK). Erythrocytes was separated from the plasma and washed three times with sterile phosphate buffer saline, centrifuged for 5 min at 1500 rpm. Separated erythrocytes were diluted with phosphate buffered saline (0.2M PBS, pH 7.4) to yield 4% suspension. To 2 mL of the suspension, different concentration (1 mL; 800–6.25 µg/mL) of the plant extracts were mixed and the final volume was adjusted to 5 mL using PBS and incubated at 37 °C for 5 min. Upon incubation, 0.5 mL of 3% H_2_O_2_ solution was added to induce the oxidative degradation of red blood cell membrane. The tubes were mixed gently and incubated (Model: R0001000920, UK) at 37 °C for 4 h with intermittent shaking. Subsequently, the tubes were centrifuged for 10 min at 1,500 rpm and the color density of the supernatant was determined using a spectrometer at 540 nm wavelength (Naim et al., 1976). Ascorbic acid and absence of a plant extracts was considered respectively as positive and negative controls. Antihaemolysis percentage of the extracts was calculated against the control was calculated as follows:

\begin{eqnarray*}\text{Percentage antihemolysis}=[({\text{Ab}}_{\mathrm{control}}-{\text{Ab}}_{\mathrm{sample}})/{\text{Ab}}_{\mathrm{control}}]\times 100. \end{eqnarray*}

Where, Ab_control_ was the absorbance of the control (water and erythrocytes without the extracts) and Ab_sample_ was the absorbance in the presence of extracts.

### Brine shrimp lethality assay

Brine shrimp lethality assay was conducted using *Artemia salina. Artemia* cysts was purchased from fish aquarium (Lanka aqua, Homagama) and was housed in a well aerated 1L capacity glass flask containing artificial seawater (prepared using 19 g of washed sea salt dissolving in 500 mL of water and adjusted to pH 8.5. After 48 h of incubation at the room temperatures (28 °C–30 °C) with constant exposure to florescence lamp to obtain free-swimming nauplii. Since cyst capsules floated on the surface, collecting nauplii from the bottom to ensure pure harvest. These freshly hatched nauplii were used for the bioassay.

Half dilution concentration series; 800, 400. 200, 100, 50, 25, 12.5, 6.25 µg/mL of both aqueous and methanol extraction of *P. subsersoa* were prepared in artificial seawater containing 2% Dimethyl sulfoxide DMSO (v/v). The assay was prepared using 20 mL of filtered seawater containing different concentrations of *P. suberosa* extracts and 1% of yeast extract (to feed nauplii) in well aerated (sufficient aeration was ensured) 25 mL of small beakers. Thirty nauplii were transferred to each beaker. The set up was kept for 24 h, under constant illumination of florescent lamp. After 24 h, umber of survived nauplii was counted under a dissecting microscope. Three replications were prepared for each concentration. Concentration series (6.25–800 µg/mL) of the standard potassium dichromate solution were used as the positive control while pure seawater with DMSO was used as the negative control. The death percentage (LC_50_) was determined using statistical analysis (Baravalia, Vaghasiya & Chanda, 2012). Percentage of death was calculated using the following equation (Sahgal et al., 2010).

\begin{eqnarray*}\text{Percentage death (%)}=[(\text{Total nauplii}-\text{live nauplii})/\text{total nauplii}]\times 100. \end{eqnarray*}

### Statistical analysis

Statistical comparison of the data was conducted using Minitab 14 for Windows software. Parametric one-way analysis of variance (ANOVA) followed by the Student *T* test was adopted to determine the significant differences. Data were expressed as the means ± standard error of the mean (SEM). The significant value was set at *p* ≤ 0.05. Linear regression analysis was used for the calculation of the toxic effect at different concentrations.

## Results

### Phytochemical analysis

Leaves were extracted using hexane, chloroform, methanol and water. The crude extracts thus obtained were subjected to qualitative phytochemical screening studies. Alkaloids, flavonoids and saponins were present in the hexane extract and saponins and anthraquinones were present in chloroform extract. Alkaloids, unsaturated sterols, triterpenes, saponins, flavonoids and tannins were observed in both methanol and water extracts. Proanthocyanidins were observed only in the aqueous extract. The phytochemical screening results are given in Table 1.

**Table 1:** Phytochemical components of chloroform, n-hexane, methanol and aqueous crude leaf extracts of *P. suberosa* leaves.

	Extraction method			
Class of compounds	Chloroform	n-hexane	Methanol	Aqueous
Alkaloids	−	+	+	+
Sterols	−	−	+	+
Titerpenes	−	−	+	+
Saponins	+	+	+	+
Flavonoids	−	+	+	+
Proanthocyanidin	−	−	−	+
Anthraquinones	+	−	−	−
Tannins	−	−	+	+

**Notes.**

+, Presence of constituent; −, Absence of constituent.

**Figure 1:**
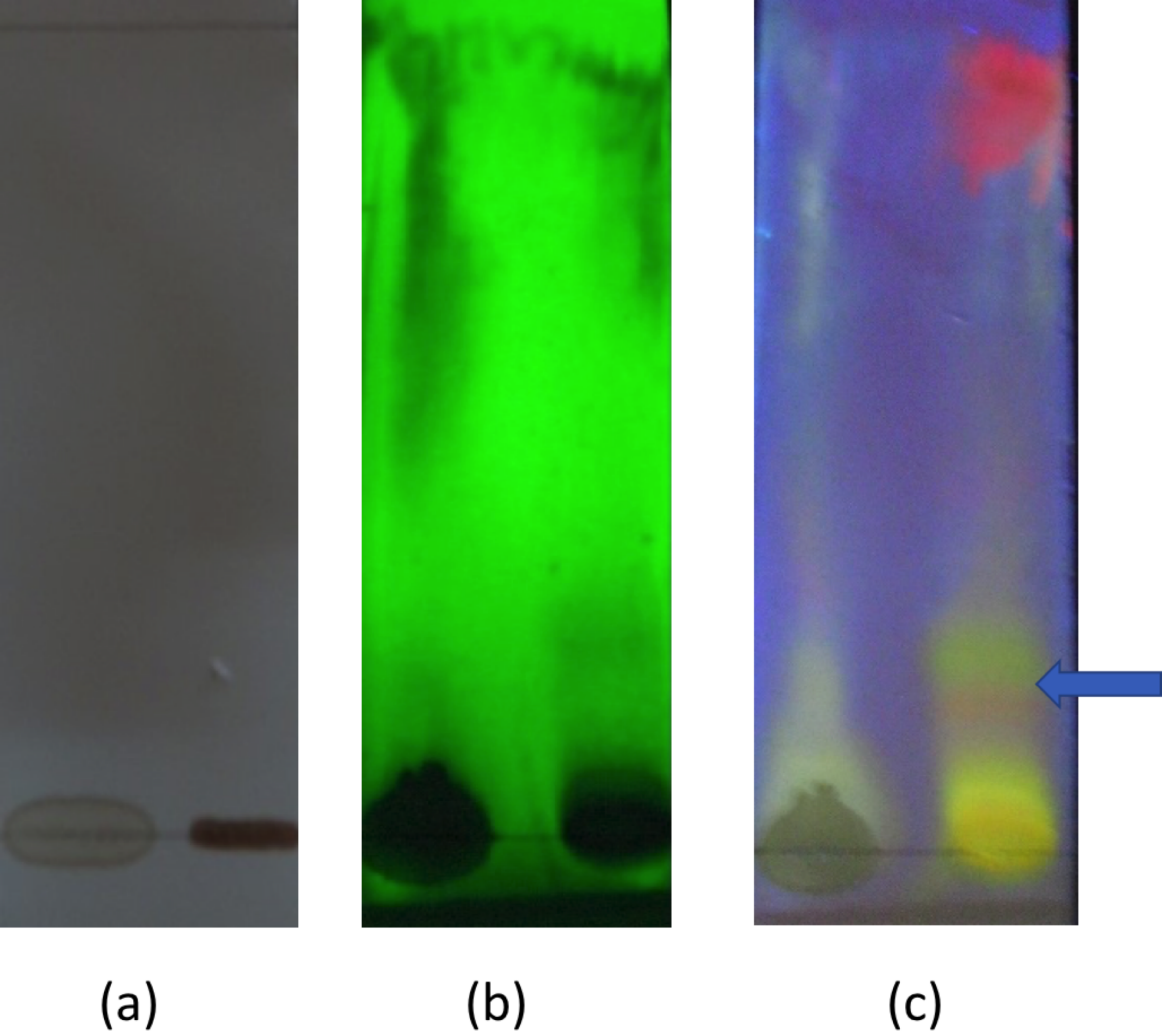
Thin layer chromatography of aqueous leaf extract of *Passiflora suberosa*. Thin layer chromatography observed under UV light (A) before spraying natural product visualizing reagent (NPR); (B) after spraying NPR, observed under UV-254 mm; and (C) after sparing NPR and observed under UV-364 mm. Orange and yellow spot appeared at UV-364 (indicated by an arrow) confirmed the presence of flavonoid.

Both flavonoids and proanthocyanidins are present in the aqueous extract. Proanthocyandins present in the aqueous extract can give a false-positive result under acidic conditions of the flavonoid test. Thus, the presence of flavonoids in the aqueous extract was confirmed by thing layer chromatographic studies using NPR as the visualizing agent. Observed fluorescence quenching at UV-254 mm and orange and yellow colour spots under UV-364 mm without and with spraying the NPR reagent respectively confirmed the presence of flavonoids in the aqueous extract (Fig. 1).

### Antibacterial screening

Antibacterial activity was evaluated in both water and methanol extracts of *P. suberosa* against three Gram-negative and three Gram-positive bacteria and the results are presented in Table 2. No activity was exhibited by the aqueous extract, while the methanol extract exhibited stronger activities against all tested bacterial strains. The methanol extract of *P. suberosa* exhibited inhibition zones ranging from 10–13 mm for Gram-negative bacteria with the greatest inhibition of zone (13 mm) produced against the clinical strain *E. coli.* Similarly, the leaf extract exhibited inhibition zones between 11–12.7 mm against Gram-positive bacteria and the maximum inhibition of 12.7 mm was observed against the pathogenic strain *S. aureus*. The positive control tested (gentamycin) exhibited an inhibition zone of 14 mm.

**Table 2:** IC_50_ values of methanol and aqueous leaf extracts from *P. suberosa*.

	Zone of inhibition (mm)		MIC (µg/mL)	
Bacterial strains	Aq	MeoH	Aq	MeoH
**Gram-positive bacteria**				
*B. subtilis*	–	10.0 ± 1.0	na	12.5
*S. aureus*	–	12.7 ± 0.6	na	25
*E. faecium*	–	11.3 ± 0.6	na	25
**Gram-negative bacteria**				
*P. aeruginosa*	–	12.3 ± 0.3	na	6.5
*S. typhimuriam*	–	12.0 ± 0	na	6.5
*E. coli*	–	13.0 ± 0.3	na	6.5

**Notes.**

Data presented as the mean ± SEM (*n* = 3). (−), no inhibition; na, not applicable; Aq, aqueous extract; MeoH, methanol extract.

The MIC assay of the methanol extract of *P. suberosa* exhibited strong activity against all 6 bacterial strains, with MIC values ranging from 6.25–25 µg/mL for different strains (Table 2). The strongest activity was exhibited against the clinical strains of Gram-negative bacteria (*E. coli*, *P. aeruginosa,* and *S. typhimuriam*) with the MIC value of 6.25 µg/mL. For the Gram-positive bacteria, the most communal MIC value was 25 µg/mL for *S. aureus* and *E. faecium* strains. Against the pathogenic strain *B. subtilis*, the MIC value detected was 12.5 µg/mL

### Antioxidant screening

The free radical- scavenging activity of DPPH was increased in a linear manner for methanol, aqueous extracts, and ascorbic acid solvent (Table 3). Aqueous extraction of leaves displays lower IC_50_ value (74.33 ± 0.88 µg/mL), than methanol extraction (418.67 ± 2.73 µg/mL), Both extracts showed significant difference (*p* < 0.05) when compared with DPPH scavenging activity value of ascorbic acid was 166.17 ± 0.60 µg/ml. Low IC_50_ value indicates a high antioxidant activity thus indicating higher antioxidant activity of the water extract than the methanol extract.

**Table 3:** The antibacterial activities of different concentrations of methanol leaf extract of *P. suberosa* against Gram positive and Gram-negative bacteria.

Experiment	Test samples	IC_50_ (µg/mL)
DPPH antioxidant activity	Methanol extract	418.67 ± 2.73
	Aqueous extract	74.33 ± 0.88
	Ascorbic acid	166.17 ± 0.60
Antihaemolytic activity	Methanol extract	610.25 ± 0.15
	Aqueous extract	80.08 ± 0.01
	Ascorbic acid	220 ± 0.01

**Notes.**

Data represented as the mean ± SEM (*n* = 9). *p < 0.05 compared with the control.

### Antihemolytic activity

Antihemolytic activities of both extracts are presented in Table 3. Both aqueous and methanol extracts of *P. suberosa* significantly (*p* < 0.05) inhibited the haemolysis of cow blood compared to positive control (ascorbic acid) in a concentration dependent manner. High antihemolytic activity was observed with the aqueous extract (IC_50_: 80.08 ± 0.01) with an inhibition value 660% greater than the methanol extract (610.25 ± 0.15).

### Brine shrimp lethality assay

**Figure 2:**
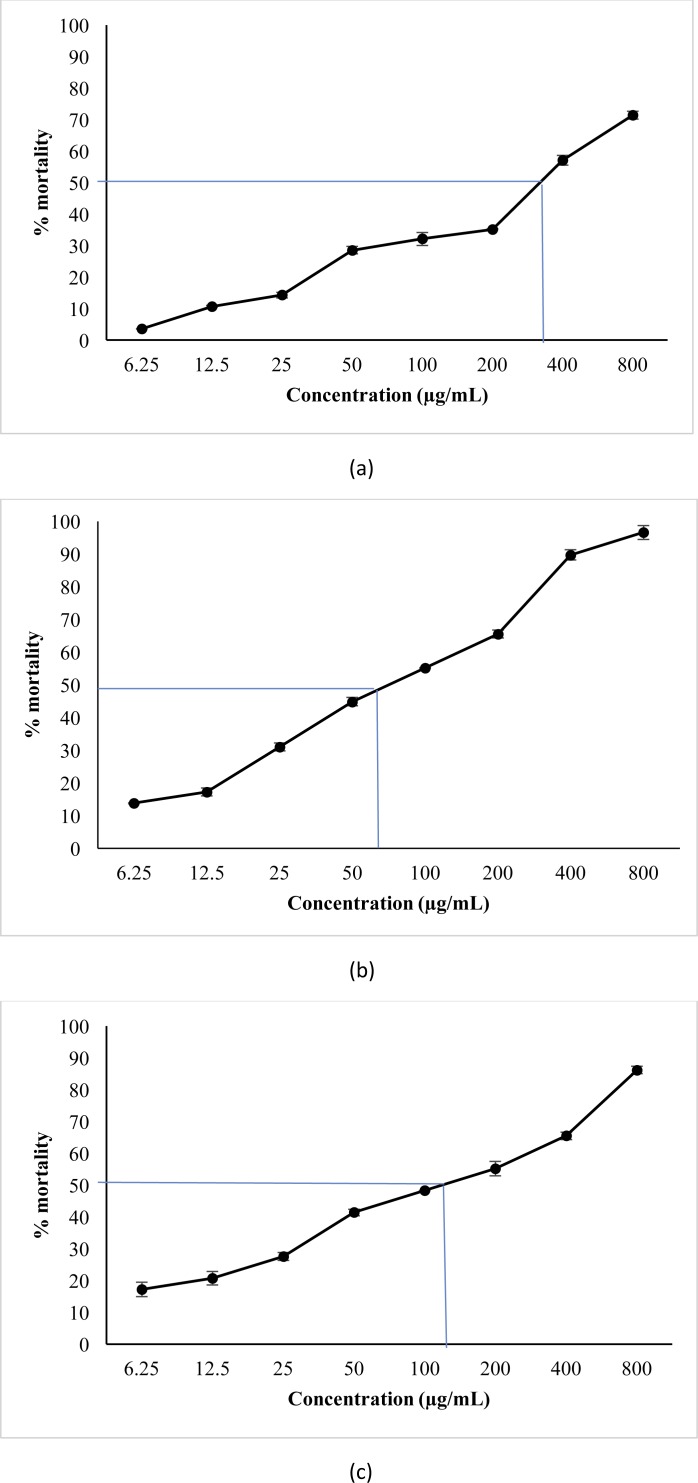
Percentage mortality of *A. salina* larvae induced by aqueous and methanol extracts of *P. suberosa* leaf at different concentrations. Methanol extract (A): IC_50_ = 309.02 ± 0.003 µg/mL; aqueous extract of *P. suberosa* leaf; (B) IC_50_ = 60.26 ± 0.80 µg/mL; potassium dichromate, positive control (C): IC_50_ = 96.31 ± 2.64 µg/mL. Data presented as the mean ± SEM (*n* = 9).

The mortality rate of brine shrimps was increased with the increasing concentrations of the both extracts (Fig. 2). This suggests a direct proportional relationship between concentrations and mortality. The highest mortality was observed in the aqueous extract. The toxic effects of both methanol and aqueous extracts showed a significant (*p* < 0.05) difference compared to the control. The IC_50_ value obtained for the water extract (60.26 ± 0.80 µg/mL) was significantly (*p* < 0.05) lower than both methanol extract (309.02 ± 0.003 µg/mL) and the positive control (96.31 ± 2.64 µg/mL) thus indicating strong activity of the aqueous extract as a toxic agent.

## Discussion

The results of our assays confirmed that *P. suberosa* is traditionally used in traditional medicinal practices to treat different disease conditions. This is the first report of *in vitro* antioxidant, cytotoxicity and antibacterial effects of *P. suberosa.* Though other species in the genus *Passiflora* are well investigated, existing knowledge of species *suberosa* is very limited.

The zones of inhibition for the methanol extraction ranged from 10–13 mm. MIC for the extract ranged from 6.25–25 µg/mL. Amongst the tested bacterial strains, *E. coli* found to be the most sensitive. Activity of crude extracts is considered to be significant if MIC values are below 100 µg/mL, moderate when 100 < MIC < 625 µg/mL or low when MIC > 625 µg/mL (Kuete, 2010). The MIC values obtained for Gram-positive and negative bacteria were equal or less than 25 µg/mL, thus indicating that the antibacterial efficacy of the methanol extract of *P. suberosa* is rather high and could be used as a potential antibacterial agent.

The data also indicated that Gram-negative bacteria were more sensitive than Gram-positive bacteria. In contrast to the previous findings of *Passiflora* species (*P. quadrangularis*, *P. maliformis*, and *P. edulis*) leaves extract with methanol, which hardly exhibited susceptible to gram negative bacteria (Ramaiya, Bujang & Zakaria, 2014), our results indicated that Gram-negative bacteria were more susceptible towards the *P. suberosa* leaf extract. The morphological difference of cell walls of Gram-positive and Gram-negative bacteria may be one of the most acceptable reason for such observation (Delcour, 2009). However, further studies on mechanisms of actions is warranted.

It has been established that tannins, saponins, phenolic compounds, and flavonoids are responsible for antibacterial potency of plants (Tiwari et al., 2011). According to phytochemical results, though both extracts of *P. suberosa* contain tannins, saponins and flavonoids, only the methanol extract showed antibacterial activities. The MIC values of conventional antibiotics are in the range of 15–107 µg/mL (Cermak et al., 2017) and observed MIC values for methanol extract of *P. suberosa* ranged from 10–13 µg/mL indicating high antibacterial activity of the plant extract. Gram-negative bacteria pose a huge challenge in modern days due to emerging drug resistance of these pathogens. The methanol extract of *P. suberosa* could be used as a potential antibacterial chemotherapy specially against Gram-negative bacteria.

Formation of reactive oxygen species (ROS) such as hydrogen peroxide, hydroxyl radical, lipid peroxides and superoxide anions occur in the body due to increased production and/or decreased elimination of ROS. The ROS production is increased during at pathophysiological condition resulting in membrane lipids, nucleic acids, carbohydrates and protein damages (Sharma et al., 2012). In the present study, the antioxidant assays conducted for both extracts resulted in a concentration-dependent protective effect against free radical generated by DPPH, which has been used as a model to investigate oxidative stress generated *in vivo* by anion radicals. Drugs such as desferoxamine inhibit generation of ROS and are capable of reducing pathological cell damages. The presence of anthocyanins, sponins and polyphenolic compounds such as flavonoids (Pandey & Rizvi, 2009) are responsible for radical scavenging by transferring protons to free radical generated by DPPH. The phytochemical screening confirms the presence of flavonoids in both extracts. The IC_50_ values obtained for the aqueous extract is six times lower than the methanol extract. Furthermore, the antioxidant capacity of the aqueous extract of *P. suberosa* species was stronger than the positive control (ascorbic acid) and antioxidant activities reported against extracts from other *Passiflora* species including *P. alata, P. edulis, P. qudraungularis,* and *P. maliformis* (Ramaiya, Bujang & Zakaria, 2014). Hence, the capacity of antioxidant efficacy of the aqueous leaf extract of *P. suberosa* leaves can be classified as strong. Only the aqueous extract showed the presence of proanthocyanidine, which may be the reason for the difference observed in the extracts.

Erythrocytes are considered as a prime target of free radical attack because they possess high amount of polyunsaturated fatty acids. Further, oxygen transport is associated with haemoglobin molecules, which are potent promoters of reactive oxygen species (Chakraborty & Shah, 2011). Both aqueous and methanol leaf extracts of *P. suberosa* were able to inhibit haemolysis of cow erythrocytes in a dose dependent manner. Presence of phenolic compounds such as flavonoids, and tannin in the extracts can be considered to be responsible for inhibition of free radicals formed as a result of lipid peroxidation (Medini et al., 2014). Antihaemolytic activity exhibited by the aqueous extract of *P. suberosa* is stronger than both methanol extract of *P. suberosa* and the positive control (ascorbic acid). Anthocyanin has been reported to reduce their haemolysis of erythrocytes due to their antioxidant efficacy (Hebbel et al., 1982). The phytochemical screening showed the presence of anthocyanin in the aqueous extract of *P. suberosa* thus leading to potent exhibited antihaemolytic activity. Furthermore, antihaemolytic activity found to be an important feature of antisickling agents. Sickle cell anemia modifies the membrane flexibility of erythrocytes making them more sensitive and fragile against free radicals. Any compound with potent antioxidant efficacy such as the aqueous extract of *P. suberosa* has the ability to prevent haemoglobin from oxidizing and thus preventing generation of free radicals (Mpiana et al., 2013) and therefore, could be used as an antisickling drug.

Brine shrimp lethality test exhibited a linear correlation between brine shrimp toxicity and human nasopharyngeal carcinoma and with some human solid tumors (Ghosh & Chatterjee, 2013). The test revealed that both methanol and aqueous extracts were capable of exhibiting mortality and toxic effects on brine shrimps Aqueous and methanol extracts of *P. suberosa* found be toxic towards Brine shrimp and the concentrations required for 50% death were 60.3 and 309 µg / mL respectively. According to the standard Brine shrimp lethality stipulation, if IC_50_ value < 100 µg/mL is considered as bioactive in toxicity evaluation of plant extracts (Meyer et al., 1982). Based on the standard benchmark, the IC_50_ value of both studied extracts showed IC_50_ value less than 400 µg/mL indicating their toxic activity. Brine shrimp toxic activity exhibited by the water extract of *P. suberosa* was about five and half times more powerful than the methanol extract. Moreover, the extracts exhibiting IC_50_ less than 100 µg/mL on Brine shrimp test is categorized as a potent toxic substance (Meyer et al., 1982). The IC_50_ value for the aqueous extract is significantly less than 100 µg/mL, thus indicating that the toxic efficacy of the aqueous extract of *P. suberosa* is quite high and could be used as a possible anticancer agent.

The Brine shrimp lethality could be related to antioxidant activity of the extracts since anthocyanins, saponins, tannins, flavonoids and polyphenols act not only as free radical scavengers but also as proliferative cell inhibitor and apoptosis inducer (Spavieri et al., 2010). The aqueous extract exhibited both high antioxidant and toxic activities when compared to the methanol extract and the respective positive controls indicating that the extract may contain high amount of phytochemicals. Therefore, the aqueous extract could have classified as a potent cytotoxic agent. The antioxidant activity of water extract of *P. suberosa* was the highest among the genus Passiflora (Ramaiya, Bujang & Zakaria, 2014).

## Conclusions

For the first time, we report the antibacterial and antioxidant activities of aqueous and methanol leaf extractions. It was discovered that the methanol extract of *P. suberosa* is a stronger antibacterial agent against Gram-negative bacteria of *P. suberosa*. We discovered a progress in the clinical development of antibacterial drug that target infectious caused by Gram-negative bacteria. The aqueous extract of *P. suberosa* exhibited strong antioxidant, antihaemolytic, and toxic activities than the methanol extract and the respective standards. Therefore, investigations with cancer cell lines are warranted to clarify anticancer properties. Isolating and examining individual bioactive compounds present in *P. suberosa* may consequently be a good candidate for the development of new drug leads against sickle cell anemia, and infectious diseases caused by Gram-negative bacteria.

##  Supplemental Information

10.7717/peerj.4804/supp-1 Figure S1 Leaves of *Passiflora suberosa* Photograph showing mature leaves, and fruits. Photo credit—Ms Hasani Sudasinghe.

10.7717/peerj.4804/supp-2 Supplemental Information 1 Raw data for antioxidant, antihemolytic and cytotoxic evaluations

## Additional Information and Declarations

### Data Availability

The following information was supplied regarding data availability: The raw data are provided in the Supplemental File.
