# Supplementary figures and images for: Potent antibacterial, antioxidant and toxic activities of extracts from Passiflora suberosa L. leaves
Source: PeerJ. 2018 May 30;6:e4804. doi: 10.7717/peerj.4804 (PMC5984578; doi:10.7717/peerj.4804)

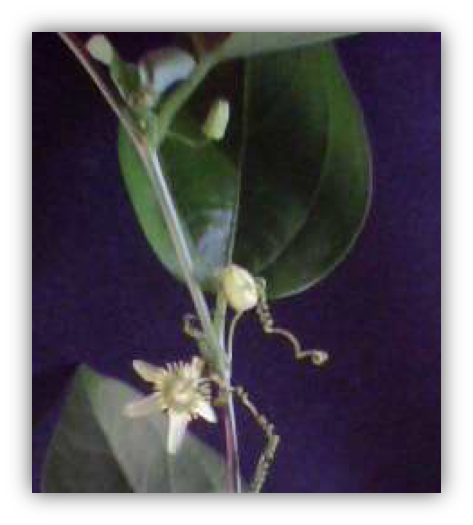

Supplement: Figure S1 — Photograph showing mature leaves, and fruits. Photo credit—Ms Hasani Sudasinghe. [file peerj-06-4804-s001.png]
