# Supplementary material for: Potent antibacterial, antioxidant and toxic activities of extracts from Passiflora suberosa L. leaves
Source: PeerJ. 2018 May 30;6:e4804. doi: 10.7717/peerj.4804 (PMC5984578; doi:10.7717/peerj.4804)
Supplement: Supplemental Information 1 [file peerj-06-4804-s002.docx]

| Different concentration series of  *Passiflora suberosa* leaf extraction from methanol  Antioxidant activity | | | | | | | | | | | | | | | | |
| --- | --- | --- | --- | --- | --- | --- | --- | --- | --- | --- | --- | --- | --- | --- | --- | --- |
| **Experiment trials** | **Absorbance Data** | **Mean values** | **Absorbance Data** | **Mean values** | **Absorbance Data** | **Mean values** | **Absorbance Data** | **Mean values** | **Absorbance Data** | **Mean values** | **Absorbance Data** | **Mean values** | **Absorbance Data** | **Mean values** | **Absorbance Data** | **Mean values** |
|  | **800 µgmL^-1^** | | **400 µgmL^-1^** | | **200 µgmL^-1^** | | **100 µgmL^-1^** | | **50 µgmL^-1^** | | **25 µgmL^-1^** | | **12.50 µgmL^-1^** | | **6.25 µgmL^-1^** | |
| Trial 1 | 0.094 | 0.10 | 0.264 | 0.256 | 0.362 | 0.365 | 0.410 | 0.415 | 0.455 | 0.452 | 0.462 | 0.464 | 0.484 | 0.481 | 0.488 | 0.486 |
|  | 0.017 |  | 0.253 |  | 0.371 |  | 0.421 |  | 0.445 |  | 0.453 |  | 0.478 |  | 0.490 |  |
|  | 0.201 |  | 0.250 |  | 0.361 |  | 0.415 |  | 0.456 |  | 0.478 |  | 0.481 |  | 0.481 |  |
| Trial 2 | 0.102 | 0.08 | 0.269 | 0.262 | 0.365 | 0.364 | 0.420 | 0.415 | 0.442 | 0.450 | 0.468 | 0.467 | 0.476 | 0.470 | 0.496 | 0.487 |
|  | 0.073 |  | 0.261 |  | 0.358 |  | 0.410 |  | 0.456 |  | 0.462 |  | 0.469 |  | 0.485 |  |
|  | 0.061 |  | 0.256 |  | 0.368 |  | 0.416 |  | 0.451 |  | 0.47 |  | 0.464 |  | 0.480 |  |
| Trial 3 | 0.108 | 0.08 | 0.265 | 0.254 | 0.351 | 0.348 | 0.408 | 0.411 | 0.444 | 0.444 | 0.468 | 0.462 | 0.478 | 0.480 | 0.469 | 0.475 |
|  | 0.020 |  | 0.245 |  | 0.352 |  | 0.416 |  | 0.439 |  | 0.452 |  | 0.484 |  | 0.477 |  |
|  | 0.106 |  | 0.251 |  | 0.342 |  | 0.410 |  | 0.448 |  | 0.465 |  | 0.477 |  | 0.478 |  |

| Different concentration series of  *Passiflora suberosa* leaf extraction from aqueous  Antioxidant activity | | | | | | | | | | | | | | | | |
| --- | --- | --- | --- | --- | --- | --- | --- | --- | --- | --- | --- | --- | --- | --- | --- | --- |
| **Experiment trials** | **Absorbance Data** | **Mean values** | **Absorbance Data** | **Mean values** | **Absorbance Data** | **Mean values** | **Absorbance Data** | **Mean values** | **Absorbance Data** | **Mean values** | **Absorbance Data** | **Mean values** | **Absorbance Data** | **Mean values** | **Absorbance Data** | **Mean values** |
|  | **800 µgmL^-1^** | | **400 µgmL^-1^** | | **200 µgmL^-1^** | | **100 µgmL^-1^** | | **50 µgmL^-1^** | | **25 µgmL^-1^** | | **12.50 µgmL^-1^** | | **6.25 µgmL^-1^** | |
| Trial 1 | 0.014 | 0.011 | 0.038 | 0.034 | 0.054 | 0.056 | 0.152 | 0.154 | 0.330 | 0.328 | 0.396 | 0.395 | 0.439 | 0.439 | 0.491 | 0.487 |
|  | 0.008 |  | 0.028 |  | 0.058 |  | 0.153 |  | 0.323 |  | 0.395 |  | 0.439 |  | 0.491 |  |
|  | 0.012 |  | 0.036 |  | 0.056 |  | 0.156 |  | 0.331 |  | 0.394 |  | 0.440 |  | 0.480 |  |
| Trial 2 | 0.023 | 0.023 | 0.045 | 0.046 | 0.062 | 0.066 | 0.170 | 0.171 | 0.332 | 0.332 | 0.411 | 0.412 | 0.448 | 0.449 | 0.465 | 0.467 |
|  | 0.027 |  | 0.045 |  | 0.067 |  | 0.178 |  | 0.332 |  | 0.412 |  | 0.448 |  | 0.468 |  |
|  | 0.019 |  | 0.049 |  | 0.069 |  | 0.165 |  | 0.333 |  | 0.412 |  | 0.450 |  | 0.468 |  |
| Trial 3 | 0.036 | 0.034 | 0.061 | 0.056 | 0.065 | 0.068 | 0.145 | 0.147 | 0.346 | 0.345 | 0.401 | 0.411 | 0.435 | 0.433 | 0.450 | 0.452 |
|  | 0.039 |  | 0.053 |  | 0.071 |  | 0.147 |  | 0.345 |  | 0.410 |  | 0.433 |  | 0.458 |  |
|  | 0.026 |  | 0.053 |  | 0.069 |  | 0.150 |  | 0.344 |  | 0.423 |  | 0.432 |  | 0.449 |  |

| Different concentration series of ascorbic acid as a positive control  Antioxidant activity | | | | | | | | | | | | | | | | |
| --- | --- | --- | --- | --- | --- | --- | --- | --- | --- | --- | --- | --- | --- | --- | --- | --- |
| **Experiment trials** | **Absorbance Data** | **Mean values** | **Absorbance Data** | **Mean values** | **Absorbance Data** | **Mean values** | **Absorbance Data** | **Mean values** | **Absorbance Data** | **Mean values** | **Absorbance Data** | **Mean values** | **Absorbance Data** | **Mean values** | **Absorbance Data** | **Mean values** |
|  | **800 µgmL^-1^** | | **400 µgmL^-1^** | | **200 µgmL^-1^** | | **100 µgmL^-1^** | | **50 µgmL^-1^** | | **25 µgmL^-1^** | | **12.50 µgmL^-1^** | | **6.25 µgmL^-1^** | |
| Trial 1 | 0.025 | 0.030 | 0.102 | 0.109 | 0.187 | 0.185 | 0.300 | 0.320 | 0.354 | 0.356 | 0.402 | 0.402 | 0.428 | 0.427 | 0.458 | 0.452 |
|  | 0.027 |  | 0.110 |  | 0.190 |  | 0.331 |  | 0.356 |  | 0.402 |  | 0.427 |  | 0.443 |  |
|  | 0.038 |  | 0.114 |  | 0.179 |  | 0.329 |  | 0.359 |  | 0.402 |  | 0.426 |  | 0.456 |  |
| Trial 2 | 0.054 | 0.050 | 0.082 | 0.072 | 0.161 | 0.178 | 0.322 | 0.328 | 0.350 | 0.347 | 0.410 | 0.413 | 0.425 | 0.430 | 0.451 | 0.444 |
|  | 0.049 |  | 0.065 |  | 0.186 |  | 0.334 |  | 0.348 |  | 0.415 |  | 0.430 |  | 0.442 |  |
|  | 0.047 |  | 0.068 |  | 0.188 |  | 0.329 |  | 0.343 |  | 0.413 |  | 0.436 |  | 0.440 |  |
| Trial 3 | 0.051 | 0.047 | 0.150 | 0.167 | 0.244 | 0.248 | 0.316 | 0.318 | 0.337 | 0.341 | 0.420 | 0.433 | 0.448 | 0.439 | 0.447 | 0.442 |
|  | 0.046 |  | 0.190 |  | 0.251 |  | 0.314 |  | 0.341 |  | 0.432 |  | 0.434 |  | 0.439 |  |
|  | 0.044 |  | 0.160 |  | 0.248 |  | 0.325 |  | 0.346 |  | 0.448 |  | 0.435 |  | 0.441 |  |

| Different concentration series of *Passiflora suberosa* leaf extraction from methanol  Cytotoxicity activity | | | | | | | | | | | | | | | | |
| --- | --- | --- | --- | --- | --- | --- | --- | --- | --- | --- | --- | --- | --- | --- | --- | --- |
| **Experiment trials** | **Death value** | **Mean values** | **Death value** | **Mean values** | **Death value** | **Mean values** | **Death value** | **Mean values** | **Death value** | **Mean values** | **Death value** | **Mean values** | **Death value** | **Mean values** | **Death value** | **Mean values** |
|  | **800 µgmL^-1^** | | **400 µgmL^-1^** | | **200 µgmL^-1^** | | **100 µgmL^-1^** | | **50 µgmL^-1^** | | **25 µgmL^-1^** | | **12.50 µgmL^-1^** | | **6.25 µgmL^-1^** | |
| Trial 1 | 9 | 9.00 | 15 | 13.00 | 19 | 18.67 | 18 | 18.33 | 16 | 20.83 | 26 | 24.33 | 26 | 24.16 | 28 | 26.83 |
|  | 7 |  | 12 |  | 17 |  | 23 |  | 21 |  | 22 |  | 26 |  | 27 |  |
|  | 9 |  | 12 |  | 20 |  | 14 |  | 24 |  | 25 |  | 19 |  | 28 |  |
| Trial 2 | 5 |  | 11 |  | 22 |  | 17 |  | 16 |  | 23 |  | 27 |  | 27 |  |
|  | 8 |  | 13 |  | 16 |  | 18 |  | 25 |  | 27 |  | 24 |  | 25 |  |
|  | 16 |  | 15 |  | 18 |  | 20 |  | 23 |  | 23 |  | 23 |  | 26 |  |

| Different concentration series of *Passiflora suberosa* leaf extraction from aqueous  Cytotoxicity activity | | | | | | | | | | | | | | | | |
| --- | --- | --- | --- | --- | --- | --- | --- | --- | --- | --- | --- | --- | --- | --- | --- | --- |
| **Experiment trials** | **Death value** | **Mean values** | **Death value** | **Mean values** | **Death value** | **Mean values** | **Death value** | **Mean values** | **Death value** | **Mean values** | **Death value** | **Mean values** | **Death value** | **Mean values** | **Death value** | **Mean values** |
|  | **800 µgmL^-1^** | | **400 µgmL^-1^** | | **200 µgmL^-1^** | | **100 µgmL^-1^** | | **50 µgmL^-1^** | | **25 µgmL^-1^** | | **12.50 µgmL^-1^** | | **6.25 µgmL^-1^** | |
| Trial 1 | 0 | 1.17 | 4 | 3.00 | 10 | 9.83 | 15 | 13.33 | 19 | 16.83 | 24 | 20.00 | 21 | 21.00 | 26 | 25.00 |
|  | 2 |  | 6 |  | 14 |  | 14 |  | 17 |  | 18 |  | 19 |  | 26 |  |
|  | 0 |  | 1 |  | 16 |  | 11 |  | 19 |  | 16 |  | 24 |  | 24 |  |
| Trial 2 | 1 |  | 0 |  | 9 |  | 10 |  | 15 |  | 20 |  | 23 |  | 22 |  |
|  | 4 |  | 3 |  | 4 |  | 16 |  | 18 |  | 21 |  | 17 |  | 27 |  |
|  | 0 |  | 4 |  | 6 |  | 14 |  | 13 |  | 21 |  | 22 |  | 25 |  |

| Different concentration series of Potassium dichromate as a positive control  Cytotoxicity activity | | | | | | | | | | | | | | | | |
| --- | --- | --- | --- | --- | --- | --- | --- | --- | --- | --- | --- | --- | --- | --- | --- | --- |
| **Experiment trials** | **Death value** | **Mean values** | **Death value** | **Mean values** | **Death value** | **Mean values** | **Death value** | **Mean values** | **Death value** | **Mean values** | **Death value** | **Mean values** | **Death value** | **Mean values** | **Death value** | **Mean values** |
|  | **800 µgmL^-1^** | | **400 µgmL^-1^** | | **200 µgmL^-1^** | | **100 µgmL^-1^** | | **50 µgmL^-1^** | | **25 µgmL^-1^** | | **12.50 µgmL^-1^** | | **6.25 µgmL^-1^** | |
| Trial 1 | 5 | 3.83 | 10 | 9.67 | 15 | 13.00 | 17 | 14.83 | 15 | 17.33 | 19 | 21.50 | 21 | 23.00 | 24 | 24.67 |
|  | 4 |  | 9 |  | 11 |  | 14 |  | 19 |  | 22 |  | 18 |  | 28 |  |
|  | 0 |  | 5 |  | 17 |  | 10 |  | 20 |  | 16 |  | 23 |  | 26 |  |
| Trial 2 | 6 |  | 11 |  | 10 |  | 18 |  | 13 |  | 25 |  | 26 |  | 19 |  |
|  | 3 |  | 13 |  | 16 |  | 17 |  | 20 |  | 21 |  | 27 |  | 26 |  |
|  | 5 |  | 10 |  | 9 |  | 13 |  | 17 |  | 26 |  | 23 |  | 25 |  |

| Different concentration series of  *Passiflora suberosa* leaf extraction from methanol  Antihemolytic activity | | | | | | | | | | | | | | | | |
| --- | --- | --- | --- | --- | --- | --- | --- | --- | --- | --- | --- | --- | --- | --- | --- | --- |
| **Experiment trials** | **Absorbance Data** | **Mean values** | **Absorbance Data** | **Mean values** | **Absorbance Data** | **Mean values** | **Absorbance Data** | **Mean values** | **Absorbance Data** | **Mean values** | **Absorbance Data** | **Mean values** | **Absorbance Data** | **Mean values** | **Absorbance Data** | **Mean values** |
|  | **800 µgmL^-1^** | | **400 µgmL^-1^** | | **200 µgmL^-1^** | | **100 µgmL^-1^** | | **50 µgmL^-1^** | | **25 µgmL^-1^** | | **12.50 µgmL^-1^** | | **6.25 µgmL^-1^** | |
| Trial 1 | 0.180 | 0.188 | 0.169 | 0.173 | 0.148 | 0.154 | 0.142 | 0.138 | 0.139 | 0.134 | 0.126 | 0.126 | 0.134 | 0.131 | 0.137 | 0.131 |
|  | 0.190 |  | 0.172 |  | 0.154 |  | 0.134 |  | 0.128 |  | 0.126 |  | 0.124 |  | 0.132 |  |
|  | 0.195 |  | 0.178 |  | 0.161 |  | 0.138 |  | 0.134 |  | 0.126 |  | 0.134 |  | 0.124 |  |
| Trial 2 | 0.179 | 0.173 | 0.164 | 0.164 | 0.176 | 0.156 | 0.156 | 0.146 | 0.146 | 0.139 | 0.138 | 0.133 | 0.122 | 0.126 | 0.114 | 0.116 |
|  | 0.172 |  | 0.158 |  | 0.146 |  | 0.144 |  | 0.138 |  | 0.132 |  | 0.126 |  | 0.119 |  |
|  | 0.167 |  | 0.171 |  | 0.146 |  | 0.138 |  | 0.133 |  | 0.129 |  | 0.130 |  | 0.114 |  |
| Trial 3 | 0.190 | 0.184 | 0.183 | 0.183 | 0.169 | 0.164 | 0.154 | 0.154 | 0.141 | 0.143 | 0.142 | 0.136 | 0.128 | 0.124 | 0.115 | 0.120 |
|  | 0.184 |  | 0.188 |  | 0.164 |  | 0.157 |  | 0.146 |  | 0.135 |  | 0.119 |  | 0.120 |  |
|  | 0.178 |  | 0.179 |  | 0.158 |  | 0.150 |  | 0.142 |  | 0.131 |  | 0.125 |  | 0.124 |  |

| Different concentration series of  *Passiflora suberosa* leaf extraction from aqueous  Antihemolytic activity | | | | | | | | | | | | | | | | |
| --- | --- | --- | --- | --- | --- | --- | --- | --- | --- | --- | --- | --- | --- | --- | --- | --- |
| **Experiment trials** | **Absorbance Data** | **Mean values** | **Absorbance Data** | **Mean values** | **Absorbance Data** | **Mean values** | **Absorbance Data** | **Mean values** | **Absorbance Data** | **Mean values** | **Absorbance Data** | **Mean values** | **Absorbance Data** | **Mean values** | **Absorbance Data** | **Mean values** |
|  | **800 µgmL^-1^** | | **400 µgmL^-1^** | | **200 µgmL^-1^** | | **100 µgmL^-1^** | | **50 µgmL^-1^** | | **25 µgmL^-1^** | | **12.50 µgmL^-1^** | | **6.25 µgmL^-1^** | |
| Trial 1 | 0.170 | 0.169 | 0.158 | 0.156 | 0.148 | 0.149 | 0.148 | 0.144 | 0.148 | 0.139 | 0.120 | 0.123 | 0.110 | 0.113 | 0.106 | 0.105 |
|  | 0.168 |  | 0.155 |  | 0.148 |  | 0.143 |  | 0.140 |  | 0.122 |  | 0.114 |  | 0.106 |  |
|  | 0.168 |  | 0.155 |  | 0.150 |  | 0.142 |  | 0.128 |  | 0.126 |  | 0.116 |  | 0.103 |  |
| Trial 2 | 0.173 | 0.174 | 0.164 | 0.162 | 0.143 | 0.142 | 0.149 | 0.139 | 0.126 | 0.130 | 0.124 | 0.118 | 0.109 | 0.109 | 0.110 | 0.107 |
|  | 0.175 |  | 0.160 |  | 0.137 |  | 0.130 |  | 0.129 |  | 0.118 |  | 0.109 |  | 0.108 |  |
|  | 0.175 |  | 0.162 |  | 0.145 |  | 0.139 |  | 0.135 |  | 0.113 |  | 0.110 |  | 0.103 |  |
| Trial 3 | 0.170 | 0.169 | 0.169 | 0.167 | 0.148 | 0.144 | 0.147 | 0.143 | 0.139 | 0.138 | 0.120 | 0.123 | 0.114 | 0.116 | 0.116 | 0.113 |
|  | 0.165 |  | 0.170 |  | 0.142 |  | 0.145 |  | 0.137 |  | 0.126 |  | 0.116 |  | 0.114 |  |
|  | 0.173 |  | 0.162 |  | 0.142 |  | 0.137 |  | 0.137 |  | 0.124 |  | 0.117 |  | 0.108 |  |

| Different concentration series of ascorbic acid as a positive control  Antihemolytic activity | | | | | | | | | | | | | | | | |
| --- | --- | --- | --- | --- | --- | --- | --- | --- | --- | --- | --- | --- | --- | --- | --- | --- |
| **Experiment trials** | **Absorbance Data** | **Mean values** | **Absorbance Data** | **Mean values** | **Absorbance Data** | **Mean values** | **Absorbance Data** | **Mean values** | **Absorbance Data** | **Mean values** | **Absorbance Data** | **Mean values** | **Absorbance Data** | **Mean values** | **Absorbance Data** | **Mean values** |
|  | **800 µgmL^-1^** | | **400 µgmL^-1^** | | **200 µgmL^-1^** | | **100 µgmL^-1^** | | **50 µgmL^-1^** | | **25 µgmL^-1^** | | **12.50 µgmL^-1^** | | **6.25 µgmL^-1^** | |
| Trial 1 | 0.429 | 0.430 | 0.401 | 0.400 | 0.391 | 0.391 | 0.365 | 0.360 | 0.327 | 0.333 | 0.282 | 0.279 | 0.271 | 0.276 | 0.234 | 0.239 |
|  | 0.431 |  | 0.402 |  | 0.394 |  | 0.359 |  | 0.335 |  | 0.282 |  | 0.277 |  | 0.241 |  |
|  | 0.431 |  | 0.397 |  | 0.389 |  | 0.357 |  | 0.336 |  | 0.274 |  | 0.281 |  | 0.241 |  |
| Trial 2 | 0.425 | 0.424 | 0.409 | 0.410 | 0.388 | 0.389 | 0.364 | 0.365 | 0.318 | 0.317 | 0.298 | 0.292 | 0.264 | 0.267 | 0.255 | 0.239 |
|  | 0.423 |  | 0.411 |  | 0.388 |  | 0.366 |  | 0.317 |  | 0.287 |  | 0.268 |  | 0.234 |  |
|  | 0.423 |  | 0.411 |  | 0.391 |  | 0.364 |  | 0.317 |  | 0.290 |  | 0.268 |  | 0.228 |  |
| Trial 3 | 0.419 | 0.423 | 0.412 | 0.412 | 0.393 | 0.393 | 0.355 | 0.354 | 0.333 | 0.328 | 0.277 | 0.279 | 0.257 | 0.250 | 0.227 | 0.220 |
|  | 0.426 |  | 0.412 |  | 0.393 |  | 0.354 |  | 0.328 |  | 0.281 |  | 0.250 |  | 0.219 |  |
|  | 0.425 |  | 0.412 |  | 0.394 |  | 0.354 |  | 0.324 |  | 0.280 |  | 0.244 |  | 0.215 |  |
